# Mr. Battley's Experiments on Aloes

**Published:** 1829-01-01

**Authors:** 


### WATERY EXTRACT OF ALOES.

Mr. Battley has made some simple experiments on the aloes vulgaris, or Barbadoes aloes, by which it appears that the cold watery solution, evaporated to the consistence of an extract, contains all the valuable part of the medicine, divested of its acrid or drastic principles. The following is the process employed.

"I.—Two pounds avoirdupois, or 2lbs. 4 oz. 2 drs. 48 grs. apothecaries' weight, of the extract of aloes, in the state in which it is imported from Barbadoes, imparted to cold distilled water 1lb. 9 oz. 1 dr. 42 grs. The solution was of a fine yellow-brown colour; its taste intensely bitter, yet free from the flavor peculiar to this species of aloe.

"II.—The residuum of I. imparted to hot distilled water 2 oz. 15 grs., leaving 4 oz. 2 drs. 15 grs. of undissolved matter. The solution was darker than I., inclining to purple, less intensely bitter, but having the peculiar flavor of the aloe vulgaris.

"III.—The residuum of II. imparted to alcohol 3 oz. 4 drs. 48 grs, leaving 5 drs. 27 grs. insoluble in this menstruum. The tincture was of a deep brown colour, bitter, and very offensive both to the taste and smell.

"IV.—The residuum of III. imparted to one part of liquor potassæ, diluted with seven parts of water, 4 drs. 48 grs., leaving V.—79 grs. of a dark earthy appearance, deprived of all aloetic properties, and void of any qualities that the taste or smell could detect. Even the alkaline solution IV. of a deep heavy brown colour, was so slightly aloetic as to be nearly insipid and inodorous.

"In I., or the cold solution of the aloe, again reduced to an extract, the profession will find at its disposal a valuable medicine; but the results II., III., will probably be found to be too drastic, and IV., V., absolutely inert."—*Journal of Morbid Anatomy.*

---

